# Baicalin Protects Against Hypertension-Associated Intestinal Barrier Impairment in Part Through Enhanced Microbial Production of Short-Chain Fatty Acids

**DOI:** 10.3389/fphar.2019.01271

**Published:** 2019-10-28

**Authors:** Dandan Wu, Liliqiang Ding, Xiaoting Tang, Wenjian Wang, Yu Chen, Teng Zhang

**Affiliations:** ^1^Clinical Research Institute of Integrative Medicine, Yueyang Hospital, Shanghai University of Traditional Chinese Medicine, Shanghai, China; ^2^Clinical Research Institute of Integrative Medicine, Shanghai Academy of Traditional Chinese Medicine, Shanghai, China

**Keywords:** baicalin, hypertension, intestinal barrier, short-chain fatty acids, gut microbiota

## Abstract

Impaired intestinal barrier plays an important role in the pathogenesis of hypertension primarily through promoting the development of chronic low-grade inflammation. Baicalin *is the major flavonoid component of Scutellaria baicalensis* Georgi, a medicinal plant commonly used for the treatment of inflammatory intestinal disorders and hypertension in traditional Chinese medicine. However, it remains to be elucidated whether baicalin alleviates hypertension-associated intestinal barrier impairment. The current study thus investigated the effects of baicalin on the intestinal barrier integrity, the intestinal expression of genes encoding proinflammatory factors and tight junction proteins, the serum levels of the inflammatory markers, the amount of fecal short-chain fatty acids (SCFAs) and the abundance of SCFAs-producing bacteria in the spontaneously hypertensive rats (SHRs). The results showed that baicalin alleviated the pathological lesions in the ilium and the proximal colon in the SHRs. Baicalin treatment resulted in decreased ileal and colonic expression of proinflammatory genes in the SHRs. In addition, baicalin treatment attenuated hypertension-associated intestinal hyperpermeability and decreased the serum levels of inflammatory indicators such as high-sensitivity C-reactive protein (hs-CRP), interleukin 1 beta, and IL-6 in the SHRs. The protective effect of baicalin on the intestinal integrity was also supported by well-preserved intestinal ultrastructure and increased intestinal expression of genes encoding tight junction proteins such as zonula occludens-1 (ZO-1), cingulin, and occludin in the SHRs. Lastly, baicalin treatment increased the amount of fecal SCFAs and the abundance of SCFAs-producing bacteria in the SHRs. In conclusion, the work here provides for the first time the morphological, biochemical, and molecular evidence supporting the protective effects of baicalin on the intestinal integrity in the SHRs, which may help better understand the therapeutic effects of *S. baicalensis* Georgi in the treatment of hypertension.

## Introduction

Hypertension is the leading risk factor for the development of cardiovascular diseases (Ahluwalia and Bangalore, 2017). Intestinal barrier impairment has recently been noted as an important pathological element implicated in the progression of hypertension (Jaworska et al., 2017; Santisteban et al., 2017). Intestinal barrier serves as a critical internal defense to restrict the systemic entry of luminal contents. Intestinal barrier impairment therefore is implicated in the pathogenesis of chronic low-grade inflammation that perpetuates the hypertensive state, exacerbates hypertensive target organ damages, and promotes the development of resistant hypertension (Fukui, 2016; Solak et al., 2016). Therapeutic agents protecting the intestinal barrier integrity under hypertensive conditions may help better control the progression of hypertension.

Baicalin is a major flavone component found in *Scutellaria baicalensis* Georgi, a medicinal herb with a long history of clinical application in the treatment of a host of diseases primarily including intestinal disorders and hypertension (Zhao et al., 2016). Anti-inflammatory and anti-hypertensive activities of baicalin have been previously documented (Zhao et al., 2016; Ding et al., 2019). However, whether baicalin is pharmacologically active at preserving the intestinal barrier integrity under hypertensive conditions remains to be investigated.

The current study thus assessed the effects of baicalin on the intestinal lesions and the intestinal expression of proinflammatory factors in SHRs. In addition, the intestinal permeability, systemic levels of hs-CRP and proinflammatory factors, the ileal and colonic ultrastructure, and the expression of genes encoding tight junction proteins were examined. Furthermore, the amount of fecal SCFAs and the abundance of SCFAs-producing bacteria in the gut were analyzed to better understand the intestinal effects of baicalin under hypertensive conditions.

## Materials and Methods

### Chemicals

Baicalin was purchased from Shanghai Yuanye Biotechnology Co., Ltd (Shanghai, China) and the purity was validated to be over 98% as previously described (Ding et al., 2019).

### Animals and Treatments

Age-matched male SHRs and Wistar-Kyoto (WKY) rats were purchased from Beijing Vital River Laboratory Animal Technology Co., Ltd. (Beijing, China). SHRs and WKY rats were housed under a controlled condition of temperature, humidity, and light (12 h light/dark cycles) and free access to food and water was allowed. The study was carried out in accordance with the recommendations of the NIH Guide for the Care and Use of Laboratory Animals. The protocol was approved by the Institutional Animal Care and Use Committee at Yueyang Hospital, Shanghai University of Traditional Chinese Medicine. Baicalin was dissolved in sterile water with the assistance of Na_2_CO_3_. SHRs were either treated with baicalin solution through oral gavage at the dose of 100 mg/kg body weight (bw) per day or received vehicle treatment in the same manner. WKY controls were gavaged with the vehicle as well. The indicated treatments were applied to 6-week animals and delivered for 6 or 14 weeks as indicated.

### Blood Pressure Measurement

The blood pressure was measured using a non-invasive tail-cuff method (Softron, Beijing, China) as previously described (Ding et al., 2019). Briefly, conscious rats were conditioned before systolic blood pressure (SBP) and diastolic blood pressure (DBP) were taken. For each measurement, at least five readings within 5–10 mm Hg range were recorded to acquire the mean of SBP and DBP.

### Histological Examination

At the end of the indicated treatments, the ileum and proximal colon specimens in the length of 3 mm were procured from the euthanized animals and subject to 4% paraformaldehyde fixation, paraffin embedding and sectioning. Paraffin sections 4 μm thick were then stained with hematoxylin and eosin (H&E) or Masson’s trichrome solution. Histopathology was recorded using a light microscope (DM2000, Leica, Germany). The number of goblet cells, the thickness of tunica muscularis, the length of the villi, and the mucosal thickness were measured after H&E staining. Masson’s trichrome positivity was quantified using Image-Pro Plus 6.

### Immunohistochemistry

At the end of the indicated treatments, the ilium and proximal colon specimens were fixed in 4% paraformaldehyde and processed for cryosectioning. Cryosections 8 μm thick were subject to immunohistochemical examination using rabbit polyclonal anti-ZO-1 (Novus Biologicals, USA) or anti-Cgn (Novus Biologicals, USA) primary antibody in conjunction with a cy3-conjugated goat-anti-rabbit secondary antibody (Sigma, USA). Counterstaining of 4-6-diamidino-2-phenylindole (DAPI) was performed to visualize the nucleus. The immunopositivity of the indicated antibody labeling was recorded by fluorescent microscopy (DM6000B, Leica, Germany).

### Transmission Electron Microscopy

To assess the intestinal ultrastructure, the ileum and the proximal colon specimens were collected after 6 weeks of the indicated treatments, fixed in 2.5% glutaraldehyde solution, followed by gradient acetone dehydration and embedding in epoxy resin. Ultrathin sections 50 nm thick were then made and subject to transmission electron microscopy (TEM) observation (Tecnai G2 Spirit Bio TWIN, FEI company, USA).

### Real-Time Reverse Transcriptase Polymerase Chain Reaction Analysis

Total RNA was isolated from the ileum and proximal colon using miRNeasy Mini Kit (Qiagen, USA), followed by reverse transcription using miScript Reverse Transcription Kit (Qiagen, USA). Real-time polymerase chain reaction (PCR) was subsequently performed to analyze the expression of genes of interest using SYBR Green PCR Master Mix (ABI, USA) on LightCycler 480 System (Roche, USA). Primer sequences are indicated in **Table 1**. Glyceraldehyde 3-phosphate dehydrogenase was included as an internal control for normalization purposes. The fold change in the gene expression was calculated according to 2^−[Ct^ ^(specific^ ^gene)−Ct^ ^(GAPDH)]^.

**Table 1:** Real-Time Reverse Transcriptase Polymerase Chain Reaction Analysis.

Gene name	Forward primer (3’-5’)	Reverse primer (3’-5’)
*Cgn*	ACTCCTGGCGAAAAGCTTCC	GTCTGCAGGTTGCTCTCAGT
*GAPDH*	ACAGCAACAGGGTGGTGGAC	TTTGAGGGTGCAGCGAACTT
*HMGB1*	TTTCCACACACCCTGCATA	GAGTCCTCAGGTAAGGAGCAG
*IL-1β*	GCTTCCTTGTGAAGTGTCT	TCTGGACAGCCCAAGTCAAG
*IL-23*	ATCTCTGCACACTAGCCTGG	ATCTTTGCAAACAGAACTGGC
*Ocln*	GATCTAGAGCCTGGAGCAACG	ATTGGGTTTGAATTCATCCGGC
*RAGE*	CTCCCTGAGGTAGGGCATGA	TCATCACCGGTTTCTGTGACC
*TLR2*	TGGAGGTCTCCAGGTCAAATCT	TTTTGCTGTGAGTCCCGAG
*TLR4*	CTCTGCCCTGCCACCATTTA	AGGAAGTACCTCTATGGGGAT
*TNF-α*	ATGGGCTCCCTCTCATCAGT	GCTTGGTGGTTTGCTACGAC
*ZO-1*	TCGAGGTCTTCGTAGCTCCA	GCAACATCAGCAATCGGTCC

### Enzyme-Linked Immunosorbent Assay

At the end of the indicated experiments, serum samples were prepared from the blood samples by centrifugation at 3,000 rpm for 10 min. The level of LBP, hs-CRP, IL-1β, and IL-6 was measured using ELISA Kits (Shanghai Yuanye Biotechnology Co., Ltd, China) according to the manufacturer’s instructions. The optical density value was measured at 450 nm using a microplate reader (BioTek, USA). For each assay, a standard cure was generated to calculate the concentration of LBP, hs-CRP, IL-1β, and IL-6 in the samples.

### Gas Chromatography-Mass Spectrometry Analysis

Fecal samples were dissolved in distilled water, vortexed, and centrifuged at 10,000 rpm for 5 min. The supernatant was filtered through 0.45 μm syringe filter, followed by extraction using 50% sulfuric solution and ether. Supernatant was collected after centrifugation at 10,000 rpm for 5 min and subject to the quantification of SCFAs by GC-MS (Agilent 6890N, USA).

### Fecal 16s Recombinant Deoxyribonucleic Acid Sequencing

Fecal samples were collected from 20-week old vehicle-treated WKY, vehicle-treated SHRs, and baicalin-treated SHRs, followed by deoxyribonucleic acid (DNA) isolation using QIAamp Fast DNA Stool Mini Kit. After PCR amplification, 16S recombinant deoxyribonucleic acid (rDNA) sequencing was performed using MiSeq System (Illumina, USA). Community and phylogenesis analysis and beta analysis were performed to assess the differences in the gut microbiota among the indicated experimental groups. The project was deposited at the European Molecular Biology Laboratory database with the accession number PRJEB34365.

### Statistical Analysis

The experimental data were expressed as the mean ± S.E.M. The statistical analyses were performed using SPSS. 21.0. Data were analyzed by one-way ANOVA with Dunnett’s post-test. Statistically significance was designated if p < 0.05.

## Results

### Baicalin Treatment Attenuates the Necrotic and Ulcerative Intestinal Lesions and Impairment of the Mechanical Intestinal Barrier in the Spontaneously Hypertensive Rats

**Figure 1:**
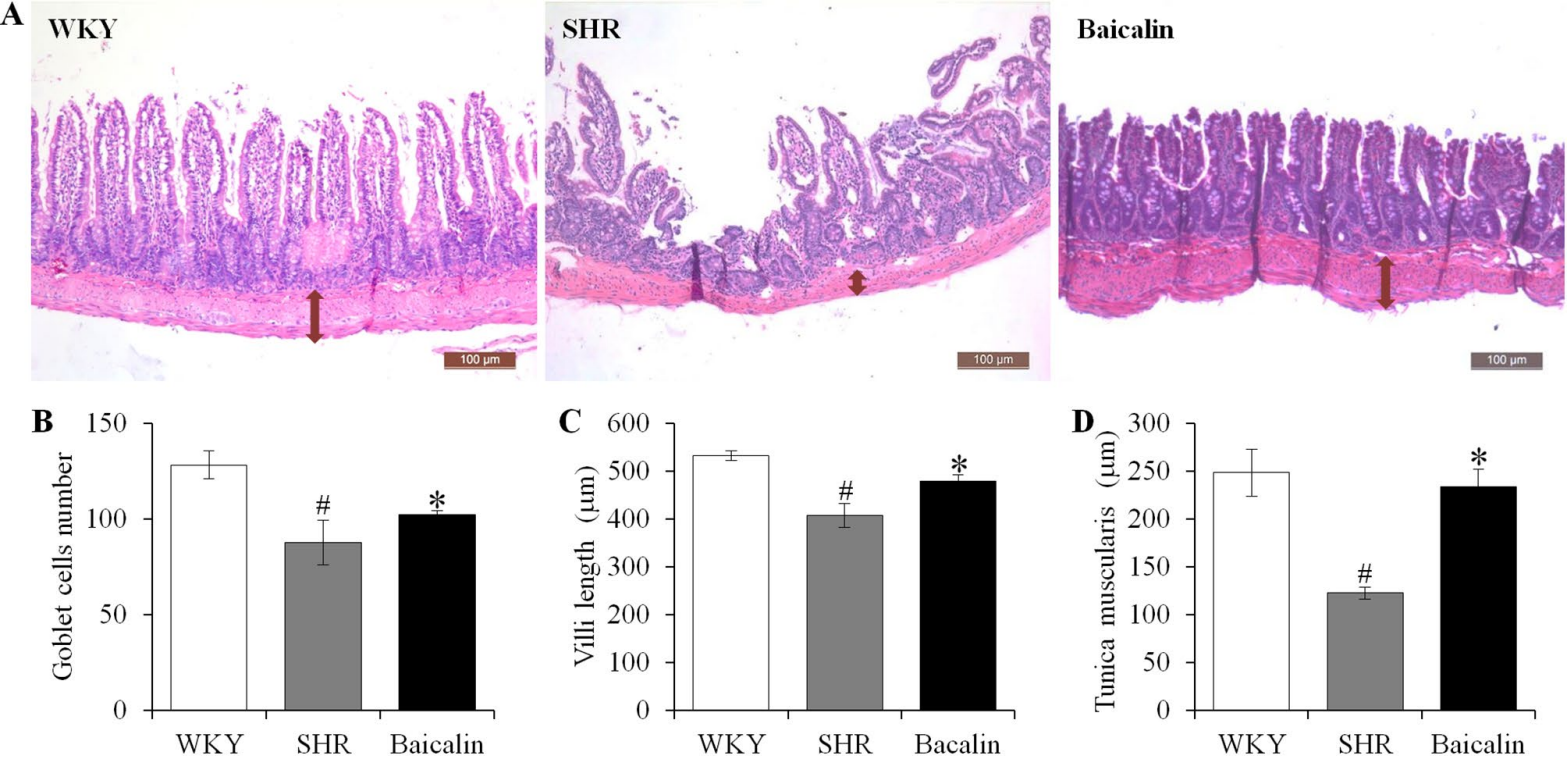
Baicalin treatment alleviates the pathological lesions in the ileum in spontaneously hypertensive rats (SHRs). The ileum specimens were collected from 12-week old vehicle-treated Wistar-Kyoto controls (WKY), vehicle-treated SHRs (SHR), and baicalin-treated SHRs (baicalin) after 6-week of the indicated treatments. The paraffin sections made from the ilium specimens were then subject to hematoxylin and eosin (H&E) staining and the ileal histology were observed and documented using a light microscope **(A)**. The number of the goblet cells **(B)** was counted and the length of the villi **(C)** and the thickness of the tunica muscularis **(D)** were measured in H&E-stained sections. Scale bar: 100 μm. Data were expressed as mean ± S.E.M (n = 6 per group). ^#^ Compared to that from the vehicle-treated WKY rats, p < 0.05; * compared to that from the vehicle-treated SHRs, p < 0.05.

**Figure 2:**
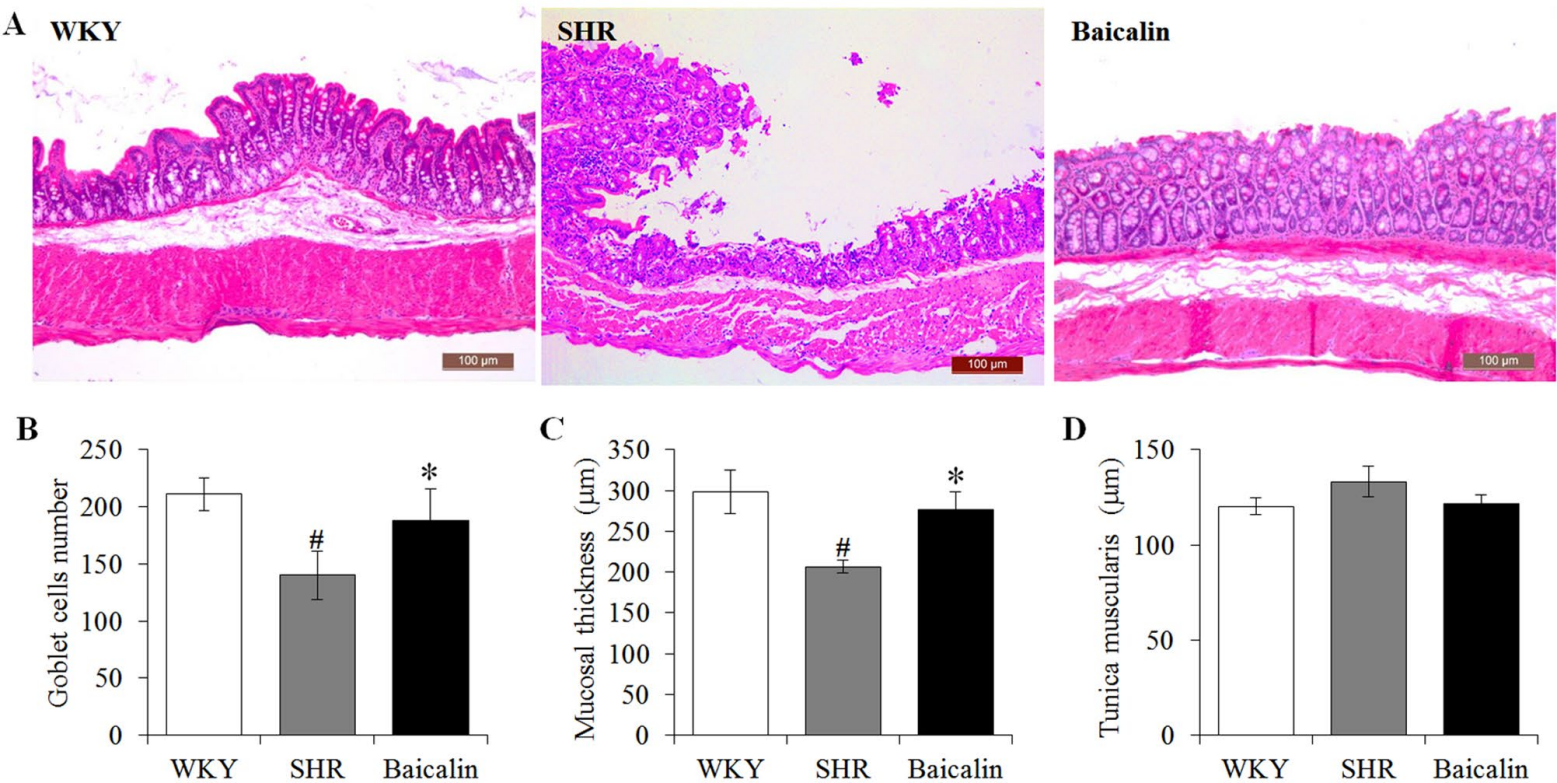
Baicalin treatment alleviates the pathological lesions in the colon in spontaneously hypertensive rats (SHRs). At the end of 6-week treatment of baicalin or vehicle, the proximal colons were collected from 12-week old vehicle-treated Wistar-Kyoto controls (WKY), vehicle-treated SHRs (SHR), and baicalin-treated SHRs (baicalin) and were processed for paraffin embedding and sectioning. Hematoxylin and eosin (H&E) staining was then performed to assess the colonic histology **(A)**. The number of the goblet cells **(B)** was counted and the thickness of the mucosal **(C)** and the tunica muscularis (D) were measured in H&E-stained sections. Scale bar: 100 μm. Data were expressed as mean ± S.E.M (n = 6 per group). ^#^ Compared to that from the vehicle-treated WKY rats, p < 0.05; * compared to that from the vehicle-treated SHRs, p < 0.05.

Anti-hypertensive effect of baicalin has been demonstrated in our previous study (Ding et al., 2019). To further investigate the impact of baicalin treatment on hypertension-associated impairment of the intestinal barrier, 6-week old SHRs were subject to a 6-week treatment regimen including either vehicle or baicalin administered at 100 mg/kg bw, a dose with a partial blood pressure-lowering effect (Ding et al., 2019), followed by histological examination of the ileal and colonic pathologies. As shown in **Supplemental Figure 1**, both SBP (**Supplemental Figure 1A**) and DBP (**Supplemental Figure 1B**) were progressively increased in the vehicle-treated SHRs compared to that from the vehicle-treated WKYs. Consistent with our previous findings (Ding et al., 2019), baicalin treatment resulted in a partial decrease in the SBP and DBP in the SHRs. In addition, as shown in **Figure 1A**, necrotic and ulcerative lesions were readily detected in the ilium in the 12-week old vehicle-treated SHRs compared to the intact ileal histology manifested by the vehicle-treated WKY controls. In contrast, baicalin treatment attenuated the ileal lesions in the SHRs (**Figure 1A**). Measurement of the number of goblet cells, villi length and the thickness of tunica muscularis further revealed that the vehicle-treated SHRs was characterized by decreased number of goblet cells, reduced villi length, and decreased thickness of tunica muscularis compared to the vehicle-treated WKY controls, whereas baicalin treatment increased the number of goblet cells, the length of the villi, and the thickness of tunica muscularis compared to that from the vehicle-treated SHRs (**Figures 1B–D**). The necrotic and ulcerative lesions were also observed in the proximal colon in the 12-week old vehicle-treated SHRs compared to that from the age-matched vehicle-treated WKY rats. Meanwhile, baicalin treatment attenuated the pathological alterations in the proximal colon in the 12-week old SHRs (**Figure 2A**). In addition, while no significant changes in the thickness of tunica muscularis were observed, the number of goblet cells and the mucosal thickness were significantly decreased in the proximal colon in the vehicle-treated SHRs compared to the vehicle-treated WKY controls. In contrast, the number of goblet cells and the mucosal thickness were significantly increased in the proximal colon in the baicalin-treated SHRs compared to their vehicle-treated SHR counterparts (**Figures 2B–D**). These results indicate that baicalin treatment ameliorates the necrotic and ulcerative intestinal lesions and impairment of the mechanical intestinal barrier in the SHRs.

### Baicalin Treatment Decreases the Ileal and Colonic Expression of Pro-Inflammatory Genes in the Spontaneously Hypertensive Rats

**Figure 3:**
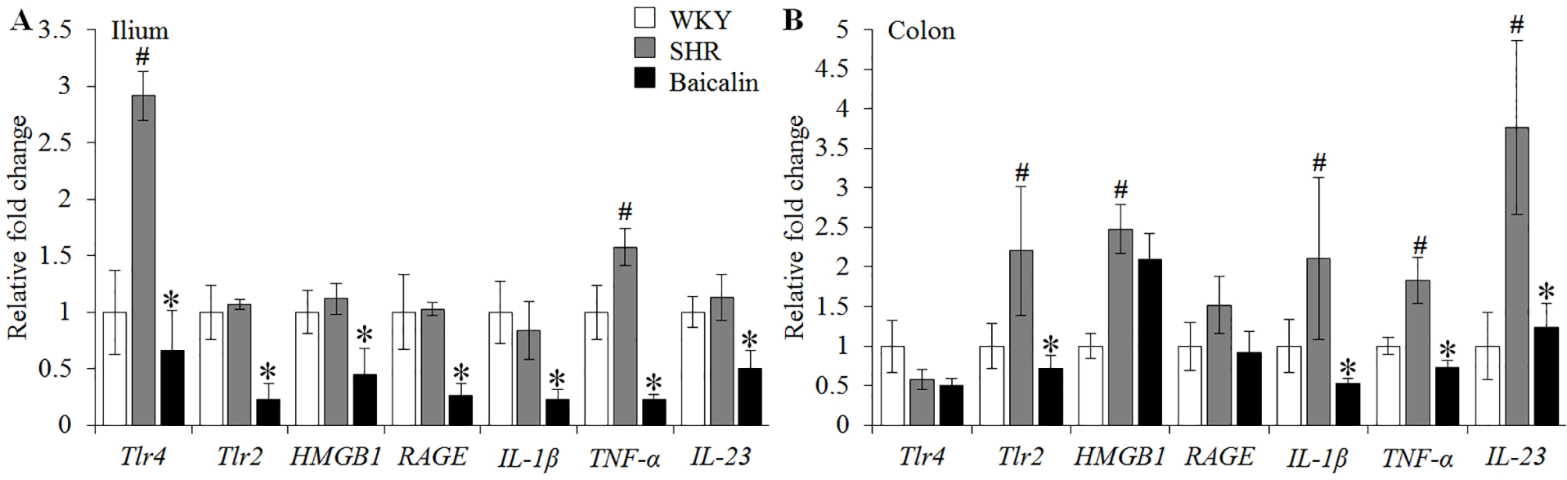
Baicalin treatment decreases the ileal and colonic expression of proinflammatory genes in spontaneously hypertensive rats (SHRs). Six-week Wistar-Kyoto (WKY) controls or SHRs received the indicated treatment for 6 weeks, followed by real-time polymerase chain reaction to analyze the ileal **(A)** and colonic **(B)** expression of *Tlr4, Tlr2, HMGB1, RAGE, interleukin 1 beta, tumor necrosis factor alpha*, and *IL-23*. Relative fold change of these genes in the vehicle-treated SHRs and baicalin-treated SHRs against that from the vehicle-treated WKYs was presented. The expression of glyceraldehyde 3-phosphate dehydrogenase was included as the internal control. Data were expressed as mean ± S.E.M (n = 6 per group). ^#^ Compared to that from the vehicle-treated WKY rats, p < 0.05; * compared to that from the vehicle-treated SHRs, p < 0.05.

Given that necrotic and ulcerative intestinal lesions were noted in the SHRs, the ileal and colonic expression of genes implicated in inflammatory responses were further analyzed, including *Tlr4, Tlr2, HMGB1, RAGE, IL-1β, TNF-α, and IL-23*. As shown in **Figure 3A**, although no significant changes in the ileal expression of *Tlr2, HMGB1, RAGE, IL-1β, and IL-23* were observed, significantly increased ileal expression of *Tlr4* and *TNF-α* was noted in the 12-week old vehicle-treated SHRs compared to that from the vehicle-treated WKY controls. In contrast, baicalin treatment resulted in significantly decreased ileal expression of not only *Tlr4* and *TNF-α*, but also *Tlr2, HMGB1, RAGE, IL-1β*, and *IL-23* in the SHRs compared to that from the vehicle-treated SHRs. Furthermore, the colonic expression of these proinflammatory genes was analyzed. As shown in **Figure 3B**, increased expression of *Tlr2, HMGB1, IL-1β, TNF-α*, and *IL-23* was observed in the 12-week old vehicle-treated SHRs compared to that from the vehicle-treated WKY controls. Baicalin treatment, however, led to significantly decreased colonic expression of *Tlr2, IL-1β, TNF-α, and IL-23* in the SHRs. These results indicate that baicalin treatment is effective at restricting the intestinal inflammatory response in the SHRs.

### Baicalin Treatment Attenuates the Fibrotic Lesions in the Ilium and the Colon in the Spontaneously Hypertensive Rats

**Figure 4:**
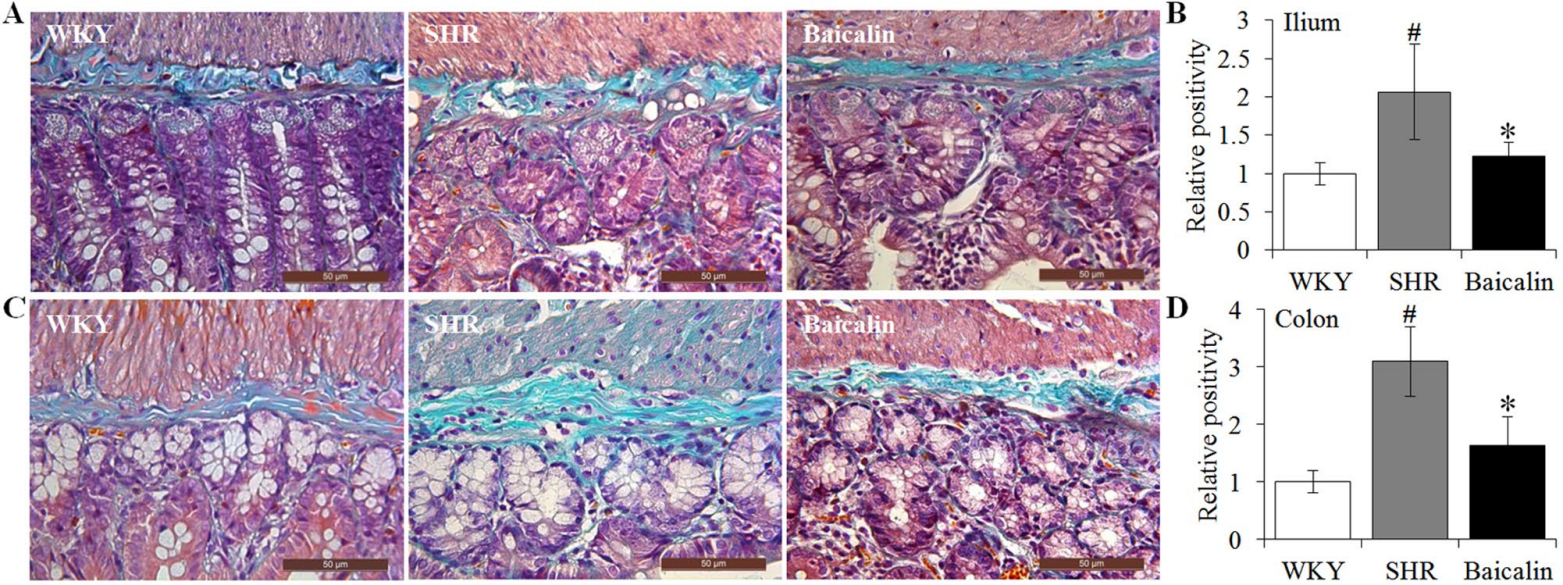
Baicalin treatment alleviates the intestinal fibrotic lesions in spontaneously hypertensive rats (SHRs). The ilium and proximal colon specimens were collected from 12-week-old vehicle-treated Wistar-Kyoto rats (WKY), vehicle-treated SHRs (SHR), and baicalin-treated SHRs (baicalin) after 6 week of the indicated treatments. Paraffin sections were then made and subject to Masson’s trichrome staining to assess the fibrotic changes in the ilium **(A)** and colon **(C)**. Masson’s trichrome positivity in the ilium **(B)** and colon sections **(D)** was then measured and expressed as the relative positivity against that from the WKY controls. Scale bar: 50 μm. Data were expressed as mean ± S.E.M (n = 6 per group). ^#^ Compared to that from the vehicle-treated WKY rats, p < 0.05; * compared to that from the vehicle-treated SHRs, p < 0.05.

Next, given that intestinal fibrotic lesions have been noted as hypertension-associated pathological changes as well (Santisteban et al., 2017), Masson’s trichrome staining, a method visualizing the collagen fibers in the tissue, was adopted to assess the effect of baicalin treatment on the fibrotic alterations in the ileum and the proximal colon in the SHRs. As shown in **Figures 4A, B**, increased Masson’s trichrome positivity was observed in the ileum in the 12-week old vehicle-treated SHRs compared to that from the vehicle-treated WKY rats, whereas baicalin treatment decreased Masson’s trichrome positivity in the ileum in the SHRs. Similar observations were made in the proximal colon. As shown in **Figures 4C, D**, increased Masson’s trichrome positivity was detected in the proximal colon in the 12-week old vehicle-treated SHRs compared to that from the vehicle-treated WKY controls, which was significantly attenuated in the baicalin-treated SHRs. These results indicate that baicalin treatment attenuates the fibrotic lesions in the ileal and proximal colon in SHRs.

### Baicalin Treatment Mitigates Intestinal Hyperpermeability and Systemic Inflammatory Response in the Spontaneously Hypertensive Rats

**Figure 5:**
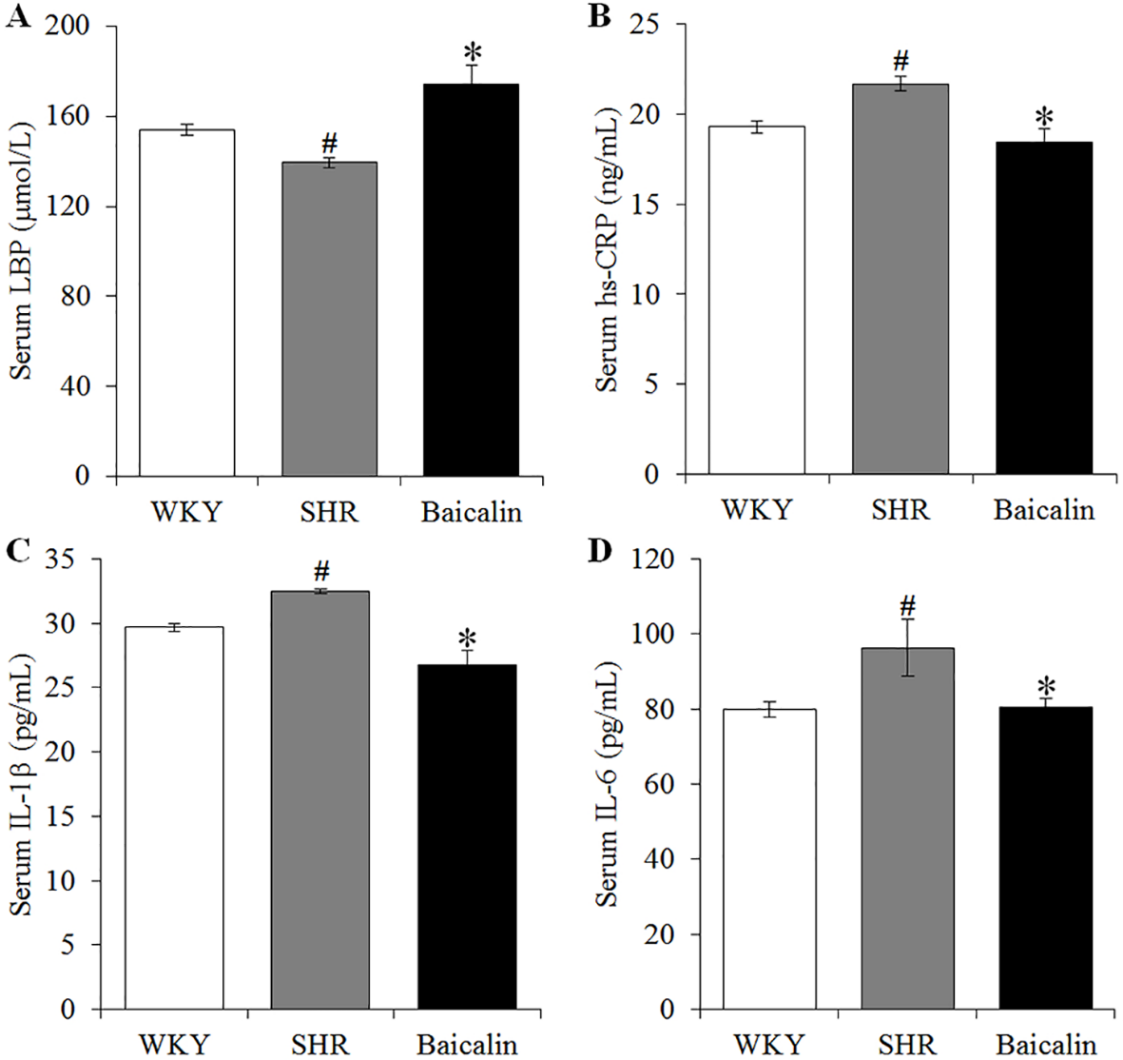
Baicalin treatment attenuates the intestinal hyperpermeability and systemic inflammatory response in the spontaneously hypertensive rats (SHRs). After 6 weeks of the indicated treatments, enzyme-linked immunosorbent assay was performed to analyze the serum levels of LBP **(A)**, high-sensitivity C-reactive protein **(B)**, interleukin 1 beta **(C)**, and IL-6 **(D)** in the vehicle-treated WKY controls (WKY), vehicle-treated SHRs (SHR) and baicalin-treated SHRs (Baicalin). Data were expressed as mean ± S.E.M (n = 10 per group). ^#^ Compared to that from the vehicle-treated WKY rats, p < 0.05; * compared to that from the vehicle-treated SHRs, p < 0.05.

Hypertension is associated with increased intestinal permeability due to impaired intestinal barrier (Jaworska et al., 2017; Santisteban et al., 2017). As shown above, baicalin treatment alleviates the pathological lesions of the mechanical intestinal barrier in the SHRs, the impact of baicalin on the intestinal permeability was further assessed. Decreased serum level of LBP serves as an indirect index of increased intestinal permeability (Gutsmann et al., 2001; Forsyth et al., 2011; Wu et al., 2019). Therefore, the serum level of LBP was analyzed to assess the effect of baicalin treatment on hypertension-associated intestinal hyperpermeability. As shown in **Figure 5A**, the serum level of LBP was found to be decreased in the 12-week old vehicle-treated SHRs compared to that from the vehicle-treated WKY controls. In contrast, baicalin treatment resulted in elevated level of serum LBP in the SHRs. Given that increased intestinal permeability is causally associated with the development of low-grade chronic inflammation, the serum levels of hs-CRP, an indicator of systemic inflammation as well as proinflammatory cytokines IL-1β and IL-6 were further analyzed. The results showed that compared to that from the vehicle-treated WKY controls, the serum levels of hs-CRP (**Figure 5B**), IL-1β (**Figure 5C**), and IL-6 (**Figure 5D**) were increased in the vehicle-treated SHRs, whereas significantly decreased serum levels of hs-CRP, IL-1β, and IL-6 were observed in the baicalin-treated SHRs. These results collectively indicate that baicalin treatment attenuates intestinal hyperpermeability and systemic inflammatory response in the SHRs.

### Baicalin Treatment Preserves the Intestinal Tight Junction in the Spontaneously Hypertensive Rats

**Figure 6:**
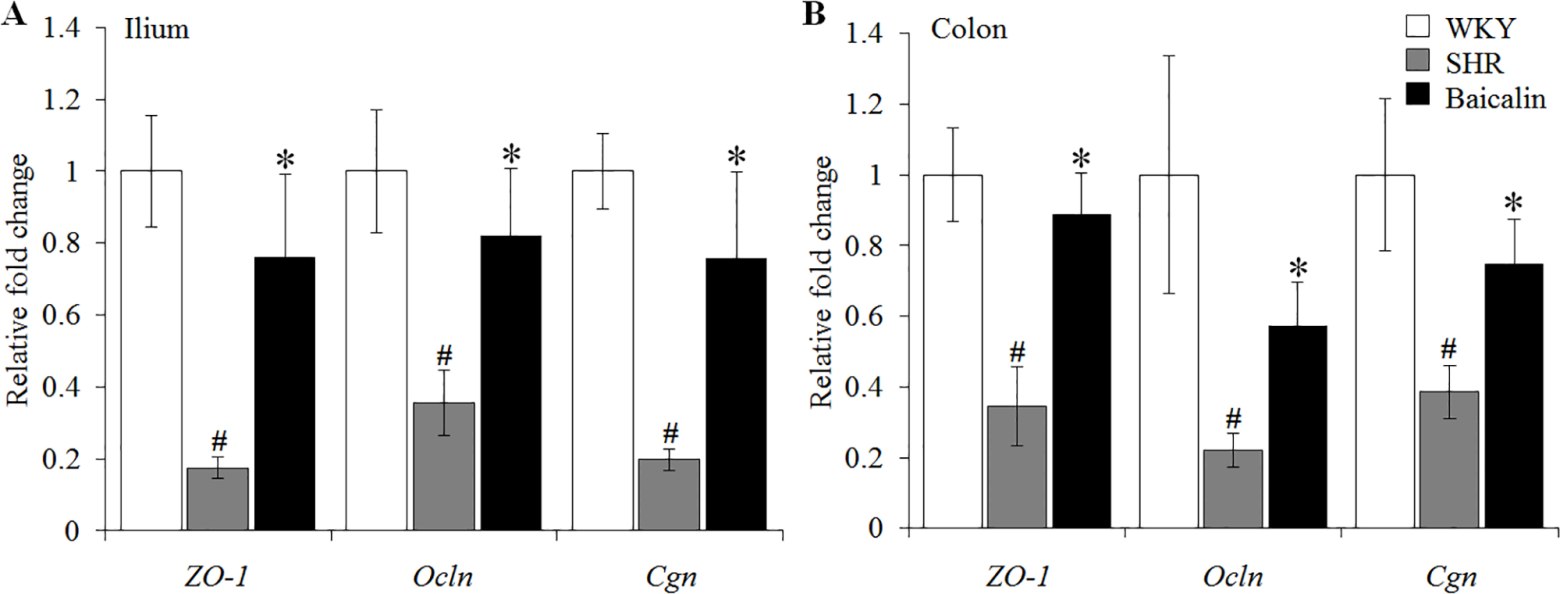
Baicalin treatment increases the ileal and colonic expression of the genes encoding zonula occludens-1 (ZO-1), Ocln, and Cgn. Real-time polymerase chain reaction was performed after 6 weeks of the indicated treatment to analyze the ileal **(A)** and colonic **(B)** expression of ZO-1, Ocln, and Cgn. The expression of glyceraldehyde 3-phosphate dehydrogenase was included as the internal control. Relative fold change of ZO-1, cingulin, and occludin in the vehicle-treated spontaneously hypertensive rats (SHRs) and baicalin-treated SHRs against that from the vehicle-treated Wistar-Kyoto (WKYs) was presented. Data were expressed as mean ± S.E.M (n = 6 per group). ^#^ Compared to that from the vehicle-treated Wistar-Kyoto rats, p < 0.05; * compared to that from the vehicle-treated SHRs, p < 0.05.

**Figure 7:**
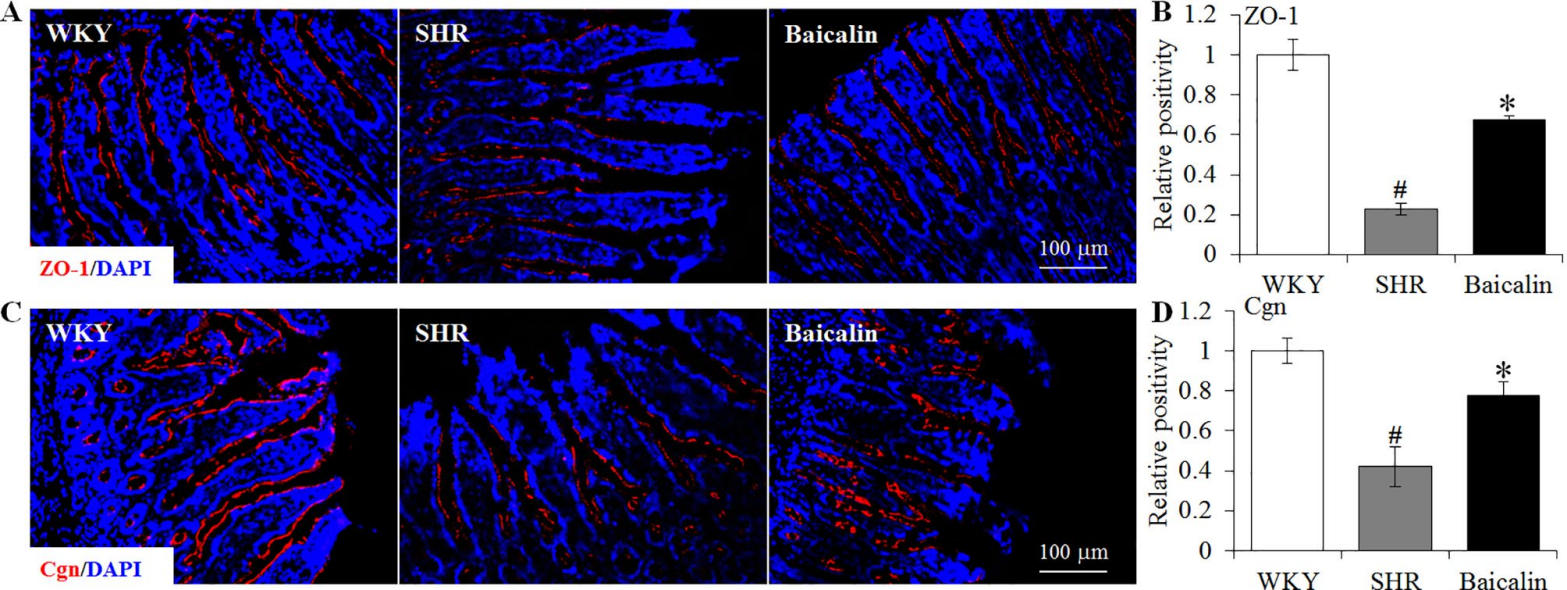
Baicalin treatment increases immunopositivity of zonula occludens-1 (ZO-1) and Cgn in the ilium in the spontaneously hypertensive rats (SHRs). Immunohistochemistry was performed at the end of the 6 weeks of the indicated treatment to reveal the expression of ZO-1 **(A)** and cingulin (Cgn) **(C)** in the ilium *in situ*. Counterstaining of 4-6-diamidino-2-phenylindole was performed to visualize the nucleus. The immunopositivity was recorded using a fluorescent microscope and analyzed using ImageJ. Relative immunopositivity of ZO-1 **(B)** and Cgn **(D)** in the vehicle-treated SHRs and baicalin-treated SHRs against that from the vehicle-treated Wistar-Kyotos (WKYs) was presented. Scale bar: 100 μm. Data were expressed as mean ± S.E.M (n = 6 per group). _#_ Compared to that from the vehicle-treated WKY rats, p < 0.05; * compared to that from the vehicle-treated SHRs, p < 0.05.

**Figure 8:**
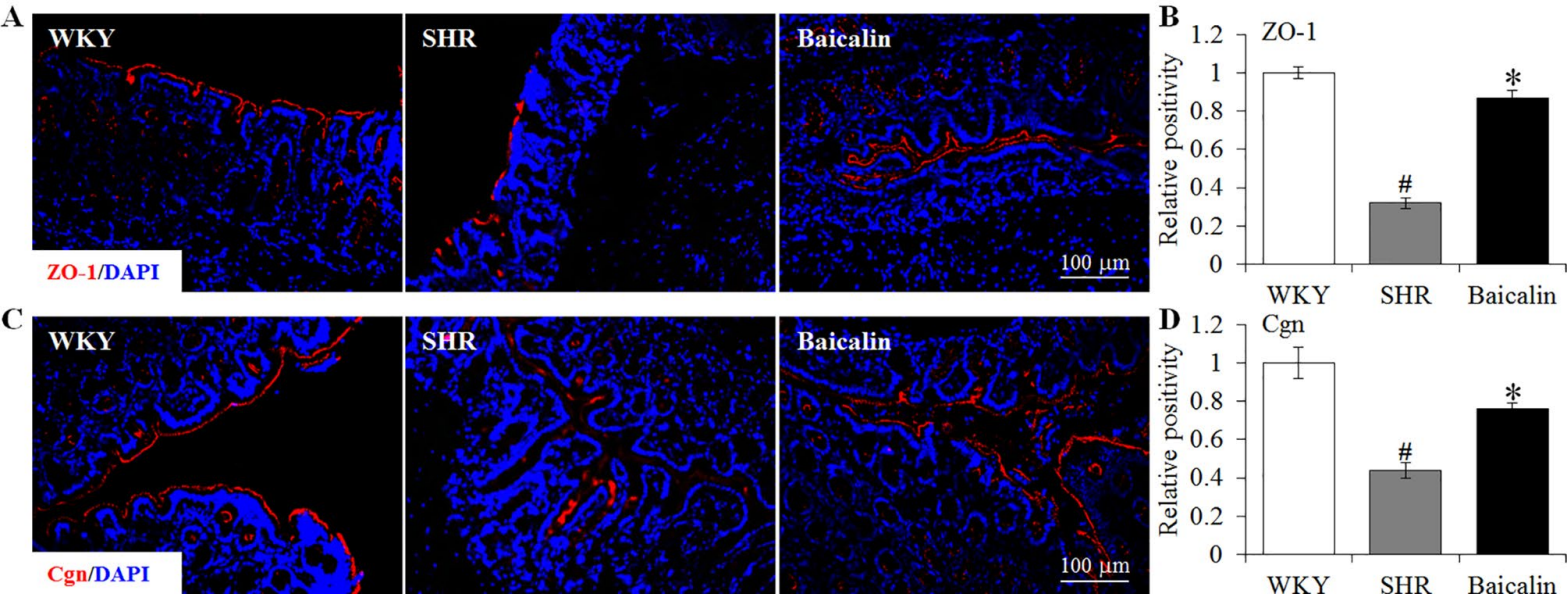
Baicalin treatment increases immunopositivity of zonula occludens-1 (ZO-1) and cingulin (Cgn) in the proximal colon in the spontaneously hypertensive rats (SHRs). Immunohistochemistry was performed at the end of the 6 weeks of the indicated treatment to reveal the expression of ZO-1 **(A)** and cingulin (Cgn) **(C)** in the proximal colon *in situ*. Counterstaining of 4-6-diamidino-2-phenylindole was performed to visualize the nucleus. The immunopositivity was recorded using a fluorescent microscope and analyzed using ImageJ. Relative immunopositivity of ZO-1 **(B)** and Cgn **(D)** in the vehicle-treated SHRs and baicalin-treated SHRs against that from the vehicle-treated Wistar-Kyoto (WKYs) was presented. Scale bar: 100 μm. Data were expressed as mean ± S.E.M (n = 6 per group). ^#^ Compared to that from the vehicle-treated WKY rats, p < 0.05; * compared to that from the vehicle-treated SHRs, p < 0.05.

**Figure 9:**
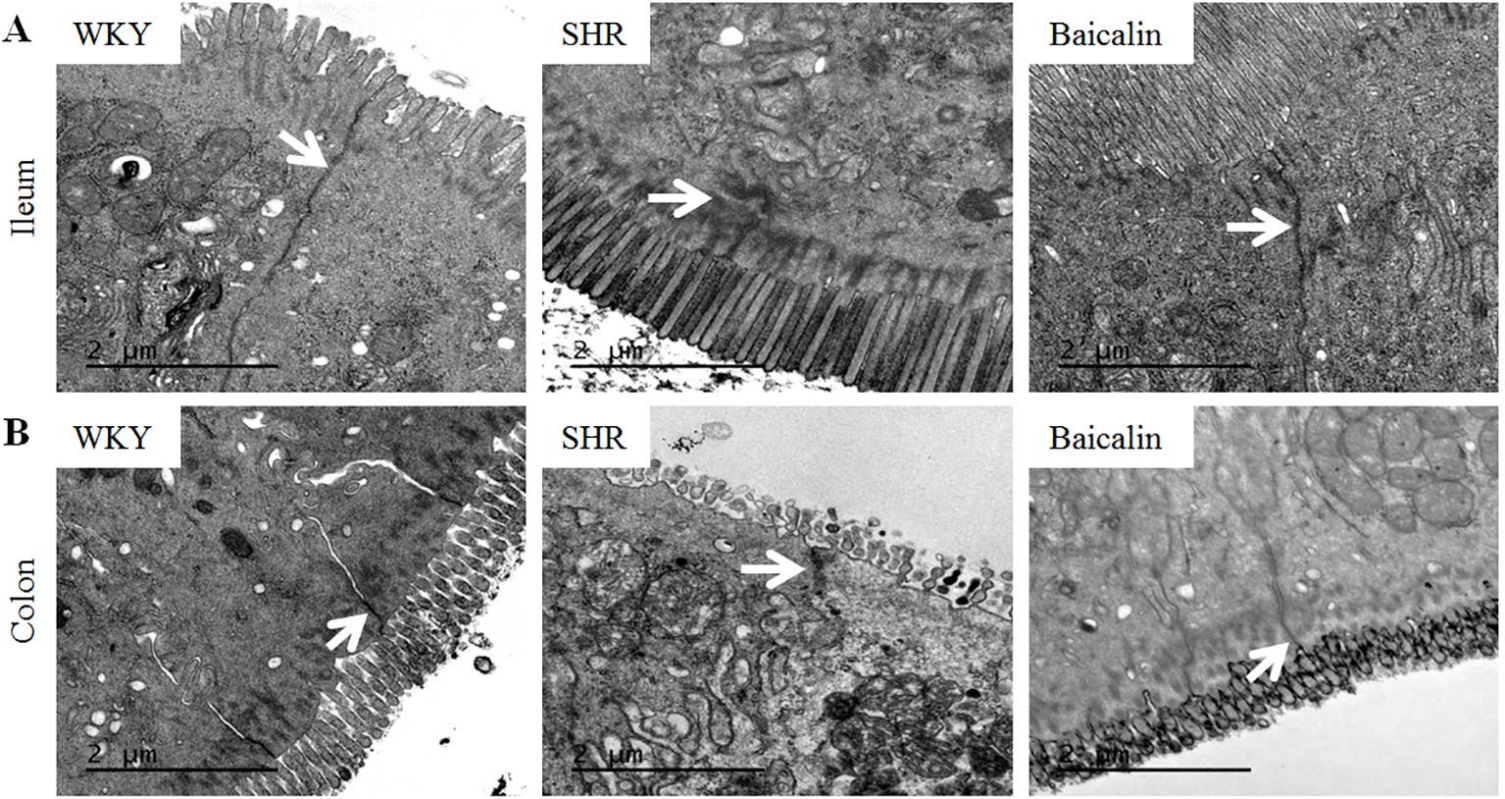
Baicalin treatment alleviates the impairment of the intestinal ultrastructure in the spontaneously hypertensive rats (SHRs). At the end of the 6-week treatment, ilium and proximal colon specimens were collected, which was followed by transmission electron microscopy to assess the changes in the ileal **(A)** and colonic **(B)** ultrastructure in the vehicle-treated Wistar-Kyoto controls (WKY), vehicle-treated SHRs (SHR), and baicalin-treated SHRs (baicalin). Scale bar: 2 μm. White arrows indicate tight junctions.

The intestinal permeability is regulated by tight junctions. Next, the ileal and colonic expression of genes encoding tight junction proteins including ZO-1, cingulin (Cgn), and occludin (Ocln) was analyzed to better understand the protective effect of baicalin against intestinal hyperpermeability in the SHRs. As shown in **Figure 6A**, compared to that from the vehicle-treated WKY rats, the ileal expression of *ZO-1*, *Cgn*, and *Ocln* was remarkably decreased in the 12-week old vehicle-treated SHRs. In distinct contrast, significantly increased ileal expression of *ZO-1*, *Cgn*, and *Ocln* was observed in the baicalin-treated SHRs compared to that from the vehicle-treated SHRs. Meanwhile, as shown in **Figure 6B**, significantly decreased colonic expression of *ZO-1*, *Cgn*, and *Ocln* was observed in the vehicle-treated SHRs compared to the vehicle-treated WKY controls. Baicalin treatment, however, resulted in increased expression of *ZO-1*, *Cgn*, and *Ocln* in the colon in the SHRs. Immunohistochemistry was also performed to visualize the changes in the intestinal tight junction proteins *in situ*. As shown in **Figures 7A, B**, decreased immunopositivity of ZO-1 was observed in the ilium in the vehicle-treated SHRs compared to that from the vehicle-treated WKY controls. On the contrary, compared to that from the vehicle-treated SHRs, increased ZO-1 immunopositivity was observed in the ilium in the baicalin-treated SHRs. In addition, baicalin treatment resulted in increased immunopositivity of Cgn in the ilium in SHRs compared to that from the vehicle-treated SHRs, in which the immunopositivity of Cgn was found to be decreased compared to the vehicle-treated WKY controls (**Figures 7C, D**). Similar observation was also made in the proximal colon (**Figure 8**). Furthermore, TEM was adopted to examine the ultrastructure of the ilium and the proximal colon. As shown in **Figure 9A**, compared to that from the vehicle-treated WKY controls, the ileal tight junction was disrupted in the vehicle-treated SHRs, which was found to be intact in the baicalin-treated SHRs. In the colonic tissue, in addition to loosened tight junction, shortened villi structure was observed in the vehicle-treated SHRs compared to that from the vehicle-treated WKYs. In contrast, baicalin treatment preserved the tight junction and the villi structure in the SHRs (**Figure 9B**). Taken together, these results indicate that baicalin treatment exerts protective effects on the intestinal integrity, in particular, intestinal tight junction under hypertensive conditions.

### Baicalin Treatment Increases the Microbial Production of Short-Chain Fatty Acids in Spontaneously Hypertensive Rats

**Figure 10:**
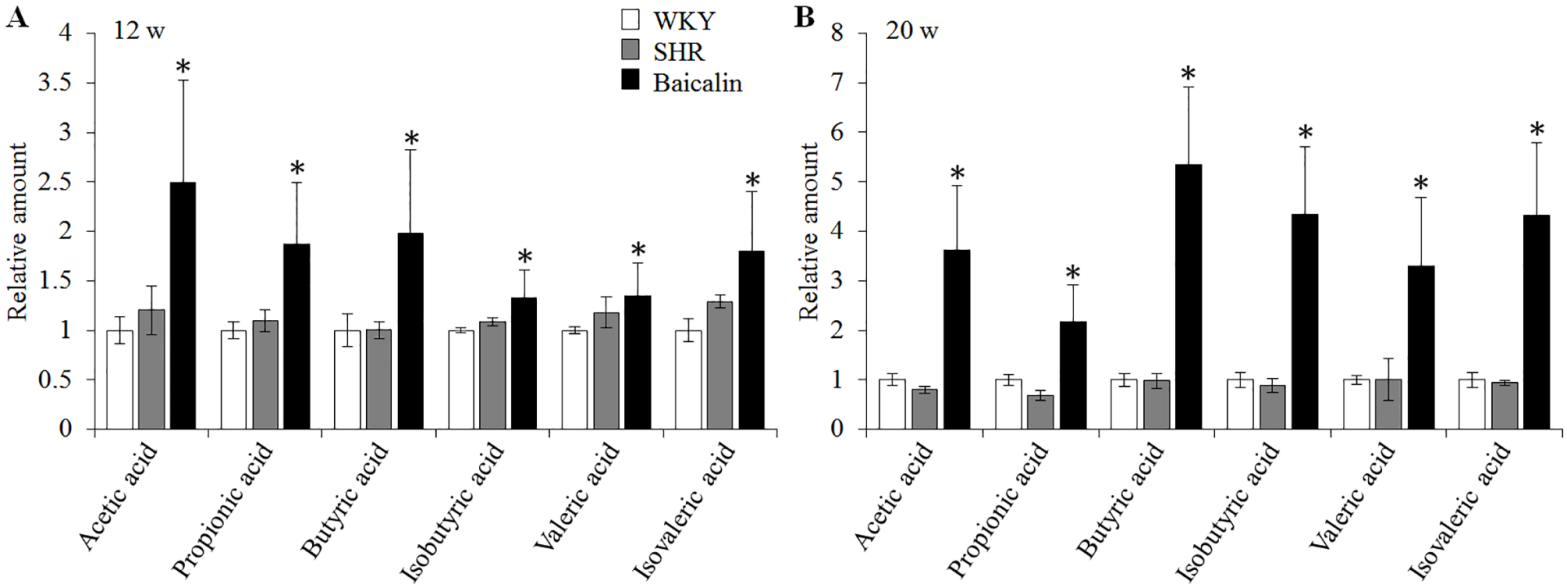
Baicalin treatment increases the fecal level of SCFAs in the spontaneously hypertensive rats (SHRs). Six-week old SHRs were treated with or baicalin for 6 weeks or 14 weeks. At the end of the indicated treatments, fecal samples were collected from 12 or 20-week old vehicle-treated Wistar-Kyoto (WKY) controls, vehicle-treated SHRs and baicalin-treated SHRs. GC-MS analyses were performed to quantify the amount of fecal SCFAs. **(A)** The relative amount of the fecal short-chain fatty acids (SCFAs) from the rats that were treated for 6 weeks. **(B)**. The relative amount of the fecal SCFAs from the rats that received the indicated treatments for 14 weeks. Data were expressed as mean ± S.E.M (n = 5 per group). * compared to that from the vehicle-treated SHRs, p < 0.05.

SCFAs are produced in the gut and are essential for the maintenance of the intestinal barrier integrity (Sakata, 1987; Peng et al., 2009). Therefore, the amount of the fecal SCFAs was further analyzed to better understand the mechanisms underlying the protective effect of baicalin on the intestinal barrier. As shown in **Figure 10A**, although no significant changes in the amount of fecal SCFAs were observed in the 12-week old vehicle-treated SHRs compared to that from the vehicle-treated WKY controls, significantly increased amount of acetic acid, propionic acid, butyric acid, isobutyric acid, valeric acid, and isovaleric acid was noted in the baicalin-treated SHRs compared to the vehicle-treated SHRs, suggesting a direct effect of baicalin on the microbial production of SCFAs in the SHRs. To validate the effect of baicalin on SCFAs production, the amount of fecal SCFAs was also analyzed in the 20-week old SHRs along with the vehicle-treated WKY controls. By 20 week of age, although without statistical significance, the amount of acetic acid and propionic acid was decreased by approximately 20 and 30%, respectively, in the vehicle-treated SHRs compared to that from the vehicle-treated WKY controls, whereas a remarkable increase in the amount of acetic acid, propionic acid, butyric acid, isobutyric acid, valeric acid, and isovaleric acid was observed in the baicalin-treated SHRs (**Figure 10B**). These results confirm that baicalin may have a direct impact on the microbial production of SCFAs in the SHRs. Thus, the abundance of SCFAs-producing bacteria was further analyzed by 16S rDNA sequencing of the fecal samples. As shown in **Table 2**, SCFAs-producing bacteria including the genus *Faecalitalea* and *Streptococcus* were not detected in the vehicle-treated SHRs compared to that from the vehicle-treated WKY controls. Baicalin treatment resulted in significantly increased abundance of *Streptococcus* in the SHRs. It is also noted that although not at a statistically significant level, the genus *Faecalitalea* was detected in the fecal samples from the baicalin-treated SHRs. In addition, the abundance of the genus *Akkermansia*, *Allobaculum*, *Bifidobacterium*, *Lachnospiraceae_NK4B4_group*, and *Roseburia* exhibited a tendency of reduction in the vehicle-treated SHRs compared to that from the vehicle-treated WKYs, in particular, the genera *Bifidobacterium* and *Lachnospiraceae_NK4B4_group* were undetectable in the vehicle-treated SHRs. However, significantly increased abundance of these genera was observed in the baicalin-treated SHRs. These results indicate baicalin treatment increases the abundance of SCFAs-producing bacteria in the SHRs.

**Table 2:** Baicalin treatment increases the abundance of short-chain fatty acids-producing bacteria in the spontaneously hypertensive rats.

Genus	Mean ± SEM			p value	
	WKY (%)	SHR (%)	Baicalin (%)	SHR *vs*. WKY	Baicalin *vs*. SHR
*Akkermansia*	0.0008 ± 0.0008	0.0030 ± 0.0030	0.0189 ± 0.0061	0.37	0.03*
*Allobaculum*	0.0257 ± 0.0114	0.0083 ± 0.0057	0.0280 ± 0.0066	0.20	0.04*
*Bifidobacterium*	0.0408 ± 0.0215	0	0.0922 ± 0.0385	0.08	0.03*
*Faecalitalea*	0.0121 ± 0.0051	0	0.003 ± 0.0014	0.03^#^	0.13
*Lachnospiraceae_NK4B4_group*	0.0023 ± 0.0015	0	0.0113 ± 0.0048	0.25	0.03*
*Roseburia*	0.1391 ± 0.0363	0.0673 ± 0.0122	0.1739 ± 0.0441	0.08	0.03*
*Ruminococcaceae_UCG-009*	0.2805 ± 0.0537	0.2238 ± 0.0425	0.375 ± 0.0403	0.50	0.02*
*Ruminococcaceae_UCG-010*	0.224 ± 0.0295	0.2495 ± 0.0693	0.4468 ± 0.0612	0.77	0.05*
*Ruminococcaceae_UCG-014*	1.5747 ± 0.0431	2.2370 ± 0.3456	3.3461 ± 0.3607	0.08	0.04*
*Ruminococcus_2*	0.0181 ± 0.0172	0.0265 ± 0.0117	0.0900 ± 0.0270	0.73	0.04*
*Streptococcus*	0.0076 ± 0.0032	0	0.0083 ± 0.0028	0.03^#^	0.01*

^#^Compared to that from the vehicle-treated WKY rats, p < 0.05; * compared to that from the vehicle-treated SHRs, p < 0.05.

## Discussion

The current study mainly demonstrates that baicalin protects against the impairment of the intestinal barrier and increases the microbial production of SCFAs under hypertensive conditions. Baicalin treatment provides partial protection against the development of hypertension-associated necrotic, ulcerative, and fibrotic changes in the intestine, suppresses the intestinal expression of proinflammatory genes, ameliorates intestinal hyperpermeability and systemic inflammation, attenuates tight junction disruption, and increases the abundance of SCFAs-producing bacteria as well as the fecal amount of SCFAs in the SHRs.

In addition to absorbing the nutrients and water, the intestine also serves as the internal barrier preventing the invasion of pathogens and limiting the systemic exposure to harmful luminal contents (Gutsmann et al., 2001; Konig et al., 2016). Disruption of the intestinal barrier increases the intestinal permeability and leads to systemic exposure of luminal endotoxins, pathogens, and antigens. This often results in altered innate immunity and triggers systemic inflammatory responses. Impaired intestinal barrier integrity is thus mechanistically implicated in the development of inflammatory state encountered in various intestinal and extra-intestinal diseases including hypertension (Cani et al., 2008; Cani et al., 2009; Fukui, 2016; Jaworska et al., 2017; Santisteban et al., 2017). Given that chronic low-grade inflammation not only is a risk factor for hypertension, but also perpetuates hypertension and promotes the development of hypertensive target organ damages and treatment resistance, maintaining the intestinal barrier integrity is important for the optimal control of hypertension. One of the major components of the intestinal barrier is the mechanical barrier primarily composed of the intestinal epithelium. The current study reveals that baicalin protects the ileal and colonic tissues from developing necrotic, ulcerative, and fibrotic lesions in the SHRs. The ileal and colonic expression of proinflammatory gene in the SHRs is decreased as a result of baicalin treatment. Meanwhile, the ultrastructure of the intestinal tight junction in the SHRs is preserved by baicalin treatment, which is paralleled by increased expression of genes encoding tight junction proteins in the intestine, decreased intestinal permeability and lower levels of inflammatory biomarkers in the serum. These results collectively support the notion that baicalin is pharmacologically active at maintaining the intestinal epithelial barrier integrity under hypertensive conditions.

The intestinal protective effects of baicalin in the SHRs may in part be attributed to its anti-inflammatory effect. Anti-inflammatory effects of baicalin have been experimentally established in different cell types. For instance, baicalin suppresses LPS-induced production of proinflammatory IL-6 and TNF-α in HBE16 airway epithelial cells (Dong et al., 2015). Baicalin inhibits TLR4 signaling in LPS-stimulated peripheral blood mononuclear cells (Ye et al., 2015). Baicalin suppresses TLR4-mediated neuroinflammation *in vivo* and in microglial cells *in vitro* (Guo et al., 2019). Baicalin also alleviates the colonic lesions in an experimental inflammatory bowel disease model *in vivo* (Zhu et al., 2016). Our current study further demonstrates that under hypertensive conditions, baicalin treatment alleviates the necrotic and ulcerative lesions and decreases the expression of proinflammatory genes in the ilium and colon. It is also noted that increased expression of proinflammatory genes in the ileum and colon appears to be affected by hypertension in a different manner. While significantly increased expression of *Tlr4* and *TNF-α* is observed in the ilium, increased expression of *Tlr2*, *HMGB1*, *TNF-α*, *IL-1β*, and *IL-23* is noted in the colon in the 12-week old SHRs. However, baicalin treatment not only counteracts hypertension-associated increase in the expression of *Tlr4* and *TNF-α* in the ilium, but also significantly decreases the ileal expression of *Tlr2*, *HMGB1*, *RAGE*, *IL-1β*, and *IL-23* in the SHRs although the expression of these genes is not altered in the vehicle-treated SHRs. In the colon, however, baicalin treatment counteracts hypertension-associated increases in the expression of *Tlr2*, *TNF-α*, *IL-1β*, and *IL-23* but not *HMGB1* in the SHRs. These results suggest that baicalin treatment may have differential impacts on the expression of proinflammatory genes in the ilium and colon. In particular, baicalin may directly decrease the basal expression of *Tlr2*, *HMGB1*, *RAGE*, *IL-1β*, and *IL-23* in the ilium, which remains to be elucidated in the future studies.

In addition, the intestines in the SHRs are characterized by disrupted intestinal tight junction structure and decreased expression of genes encoding tight junction proteins, which is attenuated as a result of baicalin treatment. These observations provide morphological and molecular evidence further supporting the protective effects of baicalin on the intestinal epithelial integrity under hypertensive conditions, which may in part result from enhanced expression of tight junction proteins as a result of baicalin treatment. In agreement with the protective effect of baicalin on the intestinal tight junctions observed in the current study, similar effect of baicalin on tight junctions has been reported by other studies. It has been demonstrated that baicalin protects against TNF-α-induced downregulation of ZO-1 expression in rat small intestinal epithelial cells *in vitro* (Wang et al., 2017). Another study has shown that baicalin restores the blood-brain barrier function through promoting the expression of tight junction proteins in cultured brain microvascular cells (Zhu et al., 2012). Therefore, the findings from our current study provide additional *in vivo* evidence supporting the protective effect of baicalin on the tight junctions. Given that baicalin treatment results in decreased intestinal expression of *TNF-α* in the SHRs, it is likely that the observed effect of baicalin on the intestinal tight junctions is in part derived from its anti-inflammatory effect.

Meanwhile, it is worth noting that baicalin treatment increases the fecal level of SCFAs under hypertensive conditions. The results from the current study also imply a direct effect of baicalin on the microbial production of SCFAs given that baicalin treatment increases the fecal level of SCFAs in the SHRs even when no significant alterations of the fecal SCFAs are noted in the vehicle-treated SHRs compared to that from the vehicle-treated WKY controls. Gut microbiota-produced metabolites play important roles in regulating normal intestinal homeostasis and multiple extra-intestinal physiological processes in the host. SCFAs, generated in the colon through microbial fermentation of undigested dietary carbohydrates, have a diverse range of health-promoting properties (Bond and Levitt, 1976). SCFAs may reduce the intestinal hyperpermeability and preserve the intestinal integrity in part through increasing the expression of tight junction proteins, thereby alleviating systemic inflammation and endotoxemia caused by intestinal hyperpermeability. For instance, as the primary energy source of colonocytes, butyrate regulates tight junctions and preserves intestinal barrier integrity (Peng et al., 2009). Butyrate also coordinates intestinal barrier protection through stabilizing hypoxia-inducible factor (Kelly et al., 2015). Relevant to the findings from the current study, increased SCFAs may in part contribute to the effect of baicalin at protecting the intestinal tight junctions and alleviating the intestinal permeability and systemic inflammation in the SHRs.

It is worth noting that the results from the current study reveal that the abundance of SCFAs-producing bacteria is increased as a result of baicalin treatment, including the genus *Roseburia*, *Ruminococcaceae UCG-009*, *Ruminococcaceae UCG-010*, *Ruminococcaceae UCG-014*, *Bifidobacterium*, *Akkermansia*, *Allobaculum*, and *Ruminococcus 2* (Ohira et al., 2017). The family *Ruminococcaceae* constitutes a type of common commensal gut bacteria that digests complex carbohydrates, serving as one of the predominant bacteria for the production of SCFAs, in particularly butyrate (Koh et al., 2016). *Bifidobacterium* spp. produces acetate and butyrate (Riviere et al., 2016). *Akkermansia*, produces butyrate and propionate (Naito et al., 2018). *Ruminococcus* spp. or *Streptococcus* generates butyrate (Riviere et al., 2016). Most members in the genus *Allobaculum* are SCFAs-producing bacteria (Everard et al., 2014). *Roseburia* spp. is among a group of dominant butyrate-producing *Firmicutes* (Takahashi et al., 2016; La Rosa et al., 2019) and plays an important role in controlling the inflammatory response in the gut primarily through the production of butyrate (Tamanai-Shacoori et al., 2017). Increased abundance of the above-mentioned SCFAs-producing bacteria in part explains elevated fecal level of SCFAs in the baicalin-treated SHRs. In addition, the effect of baicalin on increasing the abundance of SCFAs-producing bacteria and the microbial production of SCFAs is also corroborated by a recent report showing that baicalin increases the microbial production of SCFAs in mice fed with long-term high fat diet (Ju et al., 2019). However, to what extent the enhanced microbial production of SCFAs contributes baicalin-mediated intestinal barrier protection in the SHRs remains to be investigated in the future studies.

Meanwhile, vasodilatory effects of SCFAs have been reported (Mortensen et al., 1990; Nutting et al., 1991). SCFAs are involved in the blood pressure regulation *in vivo* (Pluznick, 2017). Meta-analyses have also shown that probiotic treatment or increased dietary fiber intake lowers the blood pressure presumably due to increased microbial production of SCFAs (Whelton et al., 2005; Khalesi et al., 2014). Therefore, in addition to the possible implication in baicalin-conferred intestinal barrier protection, increased production of SCFAs might in part contribute to the blood pressure-lowering effect of baicalin in the SHRs, which remains to be clarified in the future studies.

As shown in the current work, baicalin protects against hypertension-associated intestinal impairment. However, it remains to be clarified whether baicalin exerts a primary impact on the hypertensive gut or the intestinal protection occurs in a secondary manner as baicalin treatment results in a partial decrease in the blood pressure. Our results show that baicalin treatment decreases the ileal expression of *Tlr2*, *HMGB1*, *RAGE*, *IL-1β*, and *IL-23* in the SHRs when no significant changes in the expression of these genes are noted in the vehicle-treated SHRs. In addition, baicalin treatment enhances the microbial production of SCFAs prior to any hypertension-associated changes in the SCFAs are observed in the vehicle-treated SHRs. Although these results suggest a direct impact of baicalin on the hypertensive gut, it is still likely that baicalin treatment exerts a beneficial effect on the gut in part through lowering the blood pressure. Future studies are worth pursuing to clarify the intestinal effect and the associated mechanisms of baicalin under hypertensive conditions.

## Conclusion

In conclusion, the current study demonstrates for the first time that baicalin exerts protective effect on the intestinal integrity under hypertensive conditions. Most notably, baicalin treatment increases the abundance of SCFAs-producing bacteria in the gut, thereby promoting the production of SCFAs in the SHRs. Thus, the current work provides novel experimental evidence enriching the pharmacological understanding of baicalin. In addition, given that baicalin is the signature chemical component of *S. baicalensis* Georgi, the findings here may help better understand the traditional application of *S. baicalensis* Georgi in the treatment of hypertension.

## Data Availability Statement

The project was deposited at EMBL database with the accession number PRJEB34365.

## Ethics Statement

The animal study was reviewed and approved by Institutional Animal Care and Use Committee at Yueyang Hospital, Shanghai University of Traditional Chinese Medicine.

## Author Contributions

TZ and YC designed and supervised the study. DW, LD, and XT performed the experiments. DW, WW, TZ, and YC analyzed the data. TZ, DW, and YC wrote the manuscript.

## Funding

This work was supported by the Program for Professor of Special Appointment (Eastern Scholar) at Shanghai Institutions of Higher Learning (GZ2015011 and GZ2017064, TZ and YC).

## Conflict of Interest

The authors declare that the research was conducted in the absence of any commercial or financial relationships that could be construed as a potential conflict of interest.
