# Supplementary material for: Baicalin Protects Against Hypertension-Associated Intestinal Barrier Impairment in Part Through Enhanced Microbial Production of Short-Chain Fatty Acids
Source: Front Pharmacol. 2019 Oct 28;10:1271. doi: 10.3389/fphar.2019.01271 (PMC6826474; doi:10.3389/fphar.2019.01271)

Supplemental Figure. Baicalin treatment lowers the blood pressure in the SHRs. Daily baicalin treatment was orally administered to 6-week old SHRs (Baicalin) at 100 mg/kg bw for 6 weeks. Age-matched vehicle-treated SHRs (SHR) and vehicle-treated WKY rats (WKY) were included as negative and normal controls, respectively. SBP (**A**) and DBP (**B**) were measured once a week. Data were expressed as mean±SEM (n=6 per group). # Compared to that from the vehicle-treated WKYs, $p<0.05$; * compared to that from the vehicle-treated SHRs, $p<0.05$.

Supplemental Figure

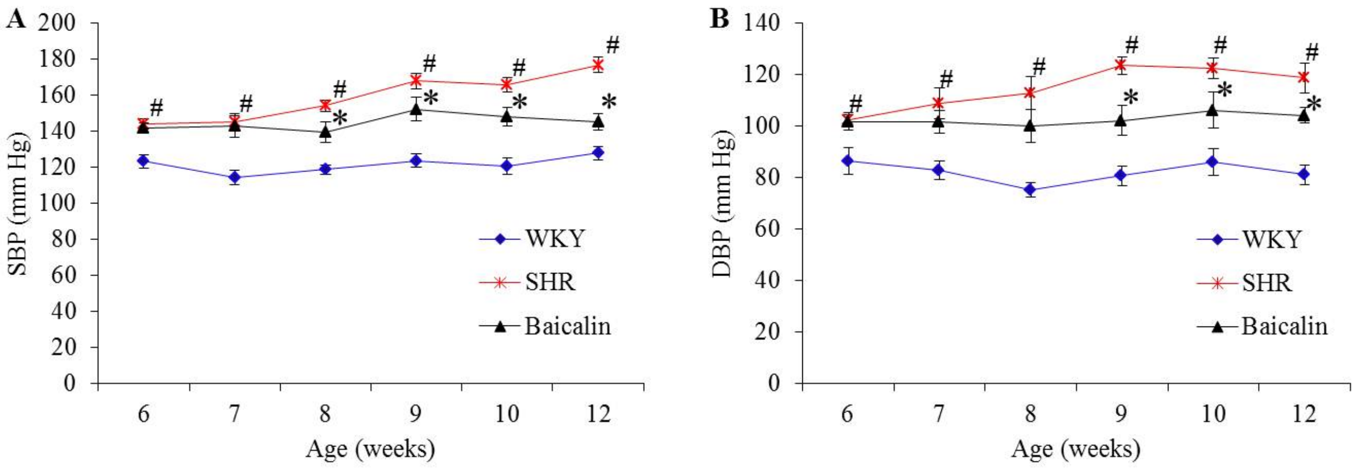

Supplement: Supplementary file 1 [file Image_1.pdf]
